# Enhancing Education and Career Pathways Through Peer and Near-Peer Social Capital

**DOI:** 10.1007/s10964-021-01540-x

**Published:** 2021-11-17

**Authors:** Ashley A. Boat, Alejandra Miranda, Amy K. Syvertsen

**Affiliations:** 1 Search Institute, Minneapolis, MN USA; 2 University of Minnesota, Minneapolis, MN USA

**Keywords:** Social capital, Peers, Near-peers, Developmental relationships, Structural equation modeling

## Abstract

Social capital strengthens emerging adults’ ability to reach life goals, but little is known about how peers and near-peers (slightly older and/or more experienced peers who serve in mentorship or coaching roles) support social capital development especially among young people of color. To address this gap, the current study examined how social capital derived from peers and near-peers contributes to emerging adults’ ability to actively mobilize social capital in pursuit of their education or career goals (i.e., self-initiated social capital) and, in turn, their education and career outcomes. A total of 841 emerging adults who participated in one of five community-based education and/or workforce support programs were surveyed (72% female; M_age_ = 20.1, *SD* = 1.84; 35% Latinx, 30% Black, 19% Asian, 16% Other). Peer social capital was indirectly associated with outcomes (i.e., progress towards education/career goals, commitment to paying-it-forward, collective efficacy to change systems) via greater self-initiated social capital, whereas near-peer social capital was both directly and indirectly associated with outcomes. The mechanisms by which peer and near-peer social capital support emerging adults as they work towards their goals may differ and have important program implications.

## Introduction

Social capital is an important vehicle for helping emerging adults make progress on the journey towards their education, career, and life goals. Social capital research has largely focused on the education and career benefits young people acquire via their parents’ social capital (Ryan, 2017). As youth transition into adulthood, however, they become increasingly autonomous and independent from their families, often turning to and relying on support from non-familial relationships such as peers and mentors. Yet, it is unclear how these relationships may support emerging adults’ social capital development as they pursue their goals. While many programs and interventions are intentionally designed to provide young adults of color with high-quality, supportive peer and/or other non-familial relationships (e.g., youth mentoring), little is known about the mechanism through which social capital derived from these relationships is associated with positive education and career outcomes and whether social capital from different types of relationships (peers vs. near-peer) equally contributes to these outcomes. To address this gap, the present study examines the extent to which peer and near-peer (slightly older and/or more experienced peers who serve in a mentorship and/or coaching role) social capital strengthens emerging adults’ self-initiated social capital (i.e., the degree to which an individual actively mobilizes their social capital to reach their goals), and in turn, their progress towards education and career goals, their commitment to helping others reach their goals, and their collective efficacy to change education and employment systems.

### Social Capital Theory

The concept of social capital exists among a wide range of disciplines and fields resulting in an equally wide range of different theoretical frameworks and conceptualizations. Across these diverse frameworks, there is universal acceptance of the premise that individuals gain access to valuable resources (e.g., information, advice, skills) through social relationships (Scales et al., 2020). Thus, social capital is a useful theory for deepening the understanding of how emerging adults access valuable relationships and resources in the navigation of postsecondary transitions. Empirical tests of social capital frameworks find positive linkages between social capital and emerging adults’ education outcomes including academic success and educational attainment (Mishra, 2020). Similarly, strong relationships within young adults’ social networks are linked with them securing their first jobs (Kramarz & Skans, 2014), and forms of social capital such as access to informal mentoring relationships are associated with a greater likelihood of securing full-time employment (McDonald et al., 2007) and obtaining more intrinsically-rewarding jobs in young adulthood (McDonald & Lambert, 2014). Because reciprocity is also an element of social capital (Torche & Valenzuela, 2011) and social capital is theorized to enhance individuals’ abilities to solve collective-action problems (Ostrom & Ahn, 2009), it is also likely that social capital positively contributes to emerging adults’ commitment to helping others reach their goals and their collective efficacy to change education and employment systems. Emerging adults who have increased social capital may feel gratitude towards their peers and therefore be more likely to pay-it-forward to others. For example, a recent systematic review showed how undocumented students utilize community resources (i.e. social capital) to overcome systemic barriers to attend higher education institutions and then went on to pay-it-forward to others through shared information and activism (Stuckey & Lambert Snodgrass, 2021). Likewise, an ethnographic study of first-generation, undocumented Latinx college students illustrated how students utilize their social capital, agency, and advocacy to build additional support services (e.g., scholarship and mentoring programs) on their college campus for their peers and subsequent generations of students (Santa-Ramirez, 2021). Studies such as these show how social capital may have ripple effects that can strengthen young adults’ collective efficacy to make positive changes to education and employment systems.

The use of social capital theory to better understand how emerging adults reach their education and career goals can be enhanced by also drawing from the field of positive youth development. This field often examines how webs of social support and/or high-quality relationships can promote positive youth outcomes (Varga & Zaff, 2018). The quality of relationships, how *developmentally* strong they are, is often understudied in social capital research (Scales et al., 2020). Yet, it is imperative to understand as relationships are malleable features of youth and young adult programs that have been empirically demonstrated to shape positive life trajectories (Chang et al., 2010). A developmental relationship—a close connection through which a young person discovers who they are, gains abilities to shape their own lives, and learns how to interact with and contribute to the world around them (Pekel et al., 2018)—may be especially important for emerging adults’ social capital. A developmental relationship is distinct from more generalized notions of positive relationships in that it is defined by the combination of five interconnected elements: express care, challenge growth, provide support, share power, and expand possibilities. The elements of a developmental relationship may provide emerging adults with more relational opportunities than other high-quality relationships by purposely providing resources such as connections to others, skill-building opportunities, and exposure to new ideas and experiences (Syvertsen et al., 2021). Informed by both social capital theory and the field of youth development, the current study defines social capital as the resources that arise from developmental relationships, which emerging adults can access and mobilize to help them improve their lives and achieve their goals (Scales et al., 2020).

### Self-Initiated Social Capital

In addition to understanding the role of developmental relationships and resources provided through these relationships, it is also important to understand the role of emerging adults’ agency in activating and extending their social capital (i.e., self-initiated social capital). Social capital is the product of two critical components: access to social capital and the use of social capital (Lin, 1999). The use of social capital requires some level of agency and/or action on the part of the individual to mobilize their social capital in pursuit of their goals. Consistent with youth systems theory (Zaff et al., 2016), young people are theorized to be active in their own development and, when given the opportunity to do so, will intentionally engage relationships and resources within their social web. Yet, few studies have examined how emerging adults activate social capital in pursuit of their goals. Therefore, the current study examines self-initiated social capital as one potential mechanism through which peer and near-peer social capital may be associated with emerging adults’ progress towards education and career goals, their commitment to helping others reach their goals, and their collective efficacy to change education and employment systems.

### Social Capital Inequities

Research shows that implicit bias, employer discrimination, and the dearth of high-quality education and employment opportunities have resulted in many young adults of color being underrepresented in postsecondary education (Levy et al., 2016) and employment pathways (Hossain & Bloom, 2015). While all emerging adults have social capital, social capital is not equitably distributed (Au, 2019). For many young adults, their school environment during adolescence provided the social and material resources needed to access education and career opportunities. School social capital is an important and valuable source of social capital; however, systemic barriers may affect the impact that school social capital has on the education and career opportunities of young people later on in life (Stephan, 2013). Due to residential segregation by race and social class, students who differ by race and socioeconomic status often have different levels of access to school social capital including less experienced teachers, fewer advanced course offerings, and fewer financial resources (Logan et al., 2012). Furthermore, teachers often underestimate the abilities of students from lower-income communities (Speybroeck et al., 2012) and demonstrate racially-based biases towards Black and Latinx students (Redding, 2019). Collectively, these experiences often limit access to and create obstacles for the development and use of social capital among emerging adults of color.

Social capital theories are useful for understanding the unequal distribution of social capital, as they assert that the more an individual can access relationships and resources, the better these resources can be purposefully mobilized by that individual (Lin, 1999). The variability in emerging adult social capital may, in turn, contribute to individual-level variation in outcomes related to postsecondary and career success. This suggests that there is a mutually reinforcing relationship between access to social capital and the use of it that directly impacts emerging adults’ education and employment opportunities. At the same time, it is also likely that some forms of social capital are more valued and/or rewarded by dominant culture than others (Carter, 2005). For example, traditional education structures (e.g., curricula, pedagogy) typically align with the needs of the dominant group and may be oppressive for students of color (Allen & White-Smith, 2018). Furthermore, forms of social and cultural capital (e.g., volunteer or internship experience, travel and/or study abroad experience, participation in extracurriculars) that privileged White, middle-class emerging adults possess (Kim & Bastedo, 2017; Lehmann, 2019) are often valued and/or rewarded in the context of postsecondary pathways more so than other, non-dominant forms of social capital (e.g., income-supporting work experience; Moreau & Leathwood, 2006). Yet, social capital derived from peers and near-peers may be an important and underutilized form of social capital that contributes to positive education and career outcomes among emerging adults of color. The social capital generated among a peer group may promote commitment to paying-it-forward and collective efforts to change education and employment systems, which over time may lead to the recognition and value of other forms of social and cultural capital that young adults of color possess.

### The Power of Peers and Near-Peers Within Relationally-Rich Organizations

Relationally-rich education and workforce support programs that serve emerging adults may be an additional or alternative resource that young adults of color can draw on to increase their access to social capital. Relationally-rich organizations are interventions and/or programs that are designed to support young people in building strong relationships with others (e.g., mentoring programs). These types of organizations can serve as brokers by connecting emerging adults to peers, mentors, coaches, potential employers, and many other social connections that may serve as developmental relationships, and increase their network of support and access to useful resources (Small, 2009). This is supported by research which shows that organizations that provide access to new and different social connections and resources (e.g., opportunities for education and career preparation, support and exploration) are effective in promoting education (Dill & Ozer, 2019), work readiness (Boat et al., 2021), and employment outcomes (Syvertsen et al., 2021). Two of the most common types of developmental relationships that emerging adults may gain access to in relationally-rich organizations are peers and near-peers.

Peers within relationally-rich workforce and education support programs can be an important source of social capital, as they often provide the emotional and social support that emerging adults require when working towards their education and career goals. Research on peer relationships has shown that strong peer relationships characterized by trust and sharing common interests has been associated with young adults’ GPA and persistence in college (Goguen et al., 2010), and that strong peer support can serve as a protective factor in the face of lower support from teachers and other adults (Brittian & Gray, 2014). Other studies show how peers can also be useful resources when seeking employment. For example, a study of 136 homeless youth and young adults (aged 13–24) found that receiving emotional support from street-peers increased the likelihood of these youth to engage in employment services (Barman-Adhikari & Rice, 2014). Similarly, another study of immigrant youth in Canada found that young people drew on their peers, rather than family members, for help with their job search (e.g., peers to act as references, share interview experiences, provide motivational support; Yan et al., 2009).

Near-peers within relationally-rich workforce and education support programs may be another critical source of social capital. Near-peers are slightly older and/or have more experience than program participants and often serve in mentorship or coaching roles. Unlike peers, these individuals may be more consistent with “institutional agents:” non-familial individuals who hold a hierarchical position of power or status and are able to provide access to highly valued resources (Stanton-Salazar, 2011; p. 1067). While near-peers may be only slightly older than program participants, it is likely they hold more positional power within the organization and have access to more personal resources that can be used to assist young people in achieving their goals (Stanton-Salazar, 2011).

Mentoring research provides some insight into the potential benefits near-peers serving in mentorship and/or coaching roles may provide. Natural mentors, for example, have been shown to play an important role in the lives of emerging adults by increasing access to education (Hurd et al., 2012) and career opportunities (Miranda-Chan et al., 2016). Like mentors, near-peers may support young adults’ postsecondary trajectories by serving as role models and by providing guidance about important life decisions (Miranda-Chan et al., 2016). Near-peers may also serve as a bridging function, by connecting emerging adults to other relationships (e.g., educators, potential employers, coworkers, classmates) and resources (e.g., how to navigate postsecondary institutions or a first job) that they otherwise would not have access to (Raposa et al., 2018). Moreover, the benefits of near-peer mentoring relationships may be especially beneficial among emerging adults of color, as interactions with natural mentors have been found to be associated with better academic (Hurd et al., 2012) and vocational outcomes (Timpe et al., 2015) among young adults from underrepresented racial/ethnic backgrounds. While support from near-peers may provide many of the same benefits of support that can come from older, adult mentors (e.g., program staff, educators), being of a similar age and having recently faced many of the same challenges, it is possible that program participants will feel a greater sense of kinship and comfort, and a smaller power differential going to near-peers for support relative to a traditional, adult mentor or educator.

## The Current Study

Based on theoretical considerations and previous empirical studies discussed in the introduction, both peer and near-peer social capital were hypothesized to be positively associated with emerging adults’ self-initiated social capital (i.e., the degree to which emerging adults actively mobilize their social capital to reach their goals), which in turn was hypothesized to be positively associated with emerging adults’ progress towards education and career goals, their commitment to helping others reach their goals, and their collective efficacy to change education and employment systems. These hypotheses were explored through a mediational model, with both peer and near-peer social capital contributing to self-initiated social capital, which in turn was conceived as the more proximal contributor to outcomes. Additional exploratory analyses further examined whether these associations varied as a function of emerging adults’ racial/ethnic identity and by the type of program emerging adults participated in (i.e., workforce vs. education support).

## Method

### Programs and Participants

Eligible participants ages 18–25 were recruited from five young adult-serving education and workforce support programs. These emerging adults and programs were recruited from a larger study, which was designed to develop, pilot test, and validate measures of social capital (Search Institute, 2021). Programs for the current study were selected to participate based on several criteria: (1) a shared mission to enhance education and/or career outcomes for young adults by strengthening program participant’s social capital, (2) programs predominately served emerging adults (ages 18–25) of color; (3) programs were designed to intentionally connect program participants with peers through a cohort model (i.e., participants experience the program with other peers and have opportunities to connect through group work and other activities) and/or with peers within their community (i.e., on their college campus), and (4) programs provide emerging adults with near-peers, individuals who serve as mentors/coaches and provide ongoing education and/or career goal support throughout the program either through face-to-face or virtual programming. Near-peers are individuals that are typically close in age to program participants or have recently gone through a similar experience as the participant (e.g., graduate of program, recently secured employment, recent college graduate). Based on these criteria, a sixth program that participated in the larger study was removed from analyses, because they served a different age range of youth (i.e., ages 14–17) and were less focused on postsecondary goals. While programs and near-peers may assist and provide support to program participants both on education and career goals, two of the programs served primarily college-aged students who may be more focused on their current education goals and three of the programs served primarily recent college graduates who are focused on securing employment. A description of each program, near-peers, and key young adult characteristics are displayed in Table 1.

**Table 1:** Characteristics of Participating Programs

Program partner	Geographic location	Program description	Near-peer description	*N*	Mage	Female gender (%)	Race/Ethnicity (%)
Program 1	New York	Workforce development program that helps first-generation college students build and leverage a professional network while building skills to manage the job search process.	Near-peers are staff members who serve as coaches and provide program participants with weekly group support calls (e.g., interview prep, LinkedIn support) and ongoing one-on-one support throughout the entire job search process. Many near-peers recently secured their first job and/or are recent alumni of the program.	105	21.8	72.6%	37.1% Latinx 32.4% Asian 17.1% Black 13.3% Other
Program 2	National	Postsecondary education support program that supports students as they navigate challenges in the classroom while also connecting them with other students and on-campus resources.	Near-peers are recent college graduates who serve as coaches. Coaches connect with students virtually through students’ preferred mode of communication and work to forge trusting one-on-one relationships by providing support and encouraging students to connect with on-campus supports (e.g., staff, health services, academic support services, affinity groups).	603	19.2	73.1%	35.5% Black 34.2% Latinx 10.5% Asian 18.5% Other
Program 3	Bay Area, Newark, Chicago, New York	Postsecondary education support program that supports students by connecting them with on-campus resources and helping them navigate the job search process upon graduation.	Near-peers are recent college graduates who serve as coaches. Coaches guide and motivate a small group of program participants (5-8 college students) weekly using a program curriculum. Coaches work with program participants individually to help them design their career vision, goals, and roadmap.	49	21.2	64.7%	40.8% Latinx 40.8% Asian 6.1% Black 12.1% Other
Program 4	Bay Area	Workforce development program that provides a learning community while teaching new tech skills and helping young adults navigate the job search process.	Near-peers are alumni of the program who serve as mentors and teachers to current program participants, as they build new skills and job search.	18	24.4	72.2%	38.9% Asian 22.2% Latinx 11.1% Black 27.8% Other
Program 5	Bay Area, New York	Workforce development program that provides a learning community while teaching digital and technical skills to help young adults jumpstart careers in digital marketing, data analytics, and tech sales.	Near-peer coaches known as captains are alumni of the program. Captains work together to guide participants through 200 hours of curriculum while providing regular check-ins to provide individual support throughout the program.	70	22.9	71.4%	48.6% Asian 30.0% Latinx 11.4% Black 10.0% Other

Latinx = Hispanic/Latino/a; Black = Black/African American; Asian = Asian/Pacific Islander; Other = Identified as another race/ethnicity

Of the 3251 program participants enrolled in one of the five programs in Winter 2021, 994 agreed to participate in the study and complete an online survey. To be included, program participants needed to be between the ages of 18 and 25; 153 program participants were excluded from the study because they were outside of this age range. Thus, a total of 841 emerging adults are included in the current study and subsequent analyses. Over half of the 841 participants identified as female (72.4%), 27.3% identified as male, 0.7% identified as non-binary/third gender, and 0.3% preferred to self-describe (e.g., gender fluid, they/them). Less than 1% of the sample (0.3%) identified as transgender. Age ranged from 18–25 years (*M* = 20.06; *SD* = 1.83). Roughly a third (34.8%) of the sample identified as Hispanic/Latinx, 29.3% identified as Black/African American, 19.0% identified as Asian/Pacific Islander, 10.2% identified as Multiracial, 5.2% identified as White, 0.2% identified as Native American or Alaskan Native, and 1.2% identified as another race.

### Procedures

A survey was administered by each of the five partner organizations using a standardized administration procedure. Partners invited all current program participants to take the online survey over a 2-week period between January and March 2021. Program participants completed the survey on computers or tablets using a web-based survey that was hosted via a secure data collection platform. Program staff helped facilitate program participants’ access to the online survey and were available to answer clarifying questions. The survey took participants roughly 10–15 minutes to complete. It was made clear to participants that the survey was anonymous, voluntary, and that choosing to not participate would in no way impact their relationship with the program. All participants had the opportunity to enter into a raffle for one of several $50 e-gift cards as a thank you for their participation. All research materials and procedures were reviewed and approved by an Institutional Review Board. Because program participants were 18 and older, they consented to their own participation.

### Measures

All measures were originally developed as part of a larger study focused on creating and validating measures of social capital. For information on the measure development process and the psychometric properties of all measures, please see (Social capital assessment and learning for equity measures technical manual; Search Institute, 2021).

#### Demographics

Age, gender and race/ethnicity information was collected in the online survey. Gender was recorded as *Male* (0) or *Female* (1). Due to small sample size, participants who identified as non-binary or self-described were recoded as missing. Race/ethnicity were controlled for in the analyses using dummy coded variables to represent each racial/ethnic group. To enable multigroup analysis, program participants who identified as Native American/Alaskan Native, Multiracial, White, or as another race were coded as Other. Latinx served as the reference group, as this represented the greatest number of participants (*n* = 287).

#### Program site

The five partner organizations were controlled for in the analyses using dummy coded variables to represent each of the sites. The program site with the greatest number of participants served as the reference group (*n* = 603).

#### Social capital

Program participants were asked about access to social capital from peers in their program and program near-peers separately. Both peer and near-peer social capital were assessed using two separate latent constructs created with two observed variables: developmental relationship and resources. The first observed variable captures the strength and quality of participants’ relationship with peers and near-peers in the program. Developmental relationships are operationalized by Search Institute’s Developmental Relationships Framework. The developmental relationships measure was a 5-item scale that asked participants to assess how much their program peers and program near-peers had shown them during the program that they mattered (express care), challenged them to be their best (challenge growth), helped them accomplish tasks (provide support), took their ideas seriously (share power), and introduced them to new experiences or opportunities (expand possibilities). Items were assessed on a 5-point scale ranging from *Strongly Disagree* (1) to *Strongly Agree* (5). Higher scores indicated a stronger developmental relationship (α = 0.90).

The second observed variable to capture the latent construct of social capital included a 3-item scale that captured the resources that individuals receive through these peer and near-peer relationships. Items included: “[My program peers/near-peer] provides me with useful information for pursuing my education or career goals,” “[My program peers/near-peer] supports me in developing or strengthening the skills needed to pursue my education or career goals,” and “[My program peers/ near-peer] connects me with other people who help me pursue my education or career goals.” The phrase [My near-peer] was replaced with the program-specific language used to describe program near-peers (e.g., Leadership Coach, College Coach). The phrase [My program peers] was replaced with the program-specific language used to describe the community of peers within the program (e.g., My [insert program name] peers, My college peers). Items were assessed on a 5-point scale ranging from *Strongly Disagree* (1) to *Strongly Agree* (5). Higher scores indicated more resources (α = 0.85).

#### Self-initiated social capital

Self-initiated social capital was measured with a three-item scale. Items included: “When working towards my education or career goals, I ask for help when I need it,” “I go out of my way to meet new people in order to reach my education or career goals,” and “I form strong relationships with people who are useful for helping me reach my education or career goals.” Items were assessed on a 5-point scale ranging from *Strongly Disagree* (1) to *Strongly Agree* (5). All items were used as indicators for a latent factor (α = 0.79).

#### Progress towards education or career goals

Three items were included in this scale: “I have made a plan to reach my education or career goals,” “I am making progress towards my education or career goals,” and “I have already taken important steps towards pursuing my education or career goals.” Items were assessed on a 5-point scale ranging from *Strongly Disagree* (1) to *Strongly Agree* (5). All items were used as indicators for a latent factor (α = 0.86). A fourth item, “I have already sought out people who can help me pursue my education or career goals,” was removed from this scale because it conceptually overlapped with items in the self-initiated social capital scale.

#### Commitment to paying-it-forward

Commitment to paying-it-forward is a four-item scale: “I do things to help others achieve their goals,” “I invest in people around me by helping them access valuable resources,” “I pass on my knowledge and skills to others,” and “I help others by introducing them to new people or connections.” Items were assessed on a 5-point scale ranging from *Strongly Disagree* (1) to *Strongly Agree* (5). All items were used as indicators for a latent factor (α = 0.88).

#### Collective efficacy to change systems

This 3-item scale gauges participants’ sense that by joining forces with others they could improve education and employment systems. Items include: “Working with others at [program name], we can create new education or career opportunities for people who might not have otherwise had them,” “Working with others at [program name], we can improve education or employment systems by using the resources we have gained from the program,” and “Working with others at [program name], we can increase access to education or career opportunities for other people like me.” Items were assessed on a 5-point scale ranging from *Strongly Disagree* (1) to *Strongly Agree* (5). All items were used as indicators for a latent factor (α = 0.92).

### Analytic Strategy

Initial confirmatory factor analysis (CFA) measurement models were created to test the fit of the following latent constructs: peer social capital, near-peer social capital, self-initiated social capital, progress towards education and/or career goals, commitment to paying-it-forward, and collective efficacy to change systems. Subsequently, structural equation modeling was used to test mediation models investigating the association between peer and near-peer social capital and outcomes through self-initiated social capital. Three separate models were run, each with peer and near-peer social capital positioned as independent variables, self-initiated social capital positioned as a mediating variable, and one focal education and career outcome (i.e., progress towards education or career goals, commitment to paying-it-forward, and collective efficacy to change systems) served as a dependent variable in three separate models. All models controlled for age, gender, race/ethnicity, and program. To evaluate the presence of mediation, the total effect of the independent variable (i.e., peer and near-peer social capital) on each of the dependent variables, can be apportioned into its direct effect and its indirect effect on the dependent variable through the proposed mediator. A bootstrapping method was employed to assess indirect effects. Confidence intervals were also estimated using bias-correcting bootstrapping methods with 1000 bootstrap sample draws (Williams & MacKinnon, 2008). All analyses were completed using Mplus version 8.0. The following fit indices were used to evaluate model fit: (1) comparative fit index (CFI) greater than 0.95, (2) root mean square error of approximation (RMSEA) below 0.06, and (3) standardized root mean square residual (SRMR) below 0.08, indicated good fit (Hu & Bentler, 1999).

Because the data were collected within programs, potential clustering effects were examined among the three variables used as outcomes in the structural equation models. The variation of program means across the five programs and the variation in emerging adult means within the programs were calculated using hierarchical linear models (Snijders & Bosker, 2012). The intraclass correlations (ICCs) revealed little variance at the program level for two of the outcome measures: progress towards education or career goals and commitment to paying-it-forward (ICCs = 0.00; *p* > 0.05 and 0.01; *p* > 0.05, respectively). The ICC for collective efficacy to change systems was 0.06; *p* < 0.05, which suggests that there may be slightly greater variation between programs for this outcome. Given the low variance at the program level across the outcomes and the small number of clusters (*n* = 5), it was determined that the best approach for accounting for any program effect was to include the programs as covariates in the structural equation models (Maas & Hox, 2004). To further examine potential program differences, exploratory multigroup models were specified to understand if the mediation effects operated differently across education and workforce support programs.

Exploratory multigroup analyses were conducted to test whether associations in the models differed by emerging adults’ racial/ethnic identity [Asian (*n* = 157), Latinx (*n* = 287), Black (*n* = 242) or Other (*n* = 139)] and by program type [workforce support program (*n* = 191); education support program (*n* = 650)]. To determine if there were any group differences in the mediating effect of peer and near-peer social capital, the *χ*2 difference test was used to examine a freely estimated model with a fully constrained model where all paths were constrained to be equal across groups. A significant change in *χ*2 between the unconstrained model and constrained model indicated the presence of group differences. If the unconstrained model presented a better fit, group differences were examined by constraining paths one by one to be equal across groups and then comparing to a fully unconstrained model to identify which specific parameters varied.

Of the total 841 respondents who provided survey data, 66.5% had complete data on all study variables; whereas 33.5% were missing data on at least one study variable. A missing-value analysis was conducted using SPSS version 26. The percentage of missing data on study variables ranged from 0% to 33.1%. Little’s MCAR test was conducted on all measures and showed that the pattern of missing values was missing completely at random, χ^2^ (71) = 89.67, *p* > 0.05. Missing data were therefore handled by using full information maximum likelihood (FIML) in model estimates, which allows for the use of cases with partially missing data. FIML provides accurate and unbiased parameter estimates under the assumption that data are missing at random (Johnson & Young, 2011).

## Results

Table 2 includes means, standard deviations, and bivariate correlations for all study variables. In general, peer and near-peer social capital and self-initiated social capital were positively correlated with all three outcomes. Older emerging adults reported greater peer social capital, greater commitment to paying-it-forward, and greater collective efficacy to change systems. Emerging adults who identified as Black reported higher levels of peer social capital, self-initiated social capital, progress towards education and/or career goals, and commitment to paying-it-forward.

**Table 2:** Descriptives and Bivariate Correlations between Study Variables

Variables	1	2	3	4	5	6	7	8	9	10	11	12
1. Age	1.00											
2. Sex (female)	−0.02	1.00										
3. Asian/Pacific Islander	0.22**	0.03	1.00									
4. Black/African American	−0.05	0.03	−0.31***	1.00								
5. Hispanic/Latinx	−0.09*	−0.02	−0.35***	−0.47***	1.00							
6. Other	−0.06	−0.05	−0.22***	−0.29***	−0.33***	1.00						
7. Near Peer Social Capital	0.07	0.01	0.00	0.01	0.04	−0.06	1.00					
8. Peer Social Capital	0.10*	−0.06	0.03	0.10*	0.08	−0.05	0.51***	1.000				
9. Self-Initiated Social Capital	0.03	−0.01	0.00	0.11**	−0.02	−0.11*	0.44***	0.52***	1.00			
10. Progress Towards Education or Career Goals	0.02	0.01	−0.06	0.12**	−0.04	−0.02	0.44***	0.45***	0.55***	1.00		
11. Commitment to Paying-it-Forward	0.08*	0.01	−0.04	0.14***	−0.04	−0.07	0.50***	0.53***	0.60***	0.53***	1.00	
12. Collective Efficacy to Change Systems	0.18***	0.04	−0.01	0.02	0.06	−0.08	0.65***	0.44***	0.47***	0.45***	0.59***	1.00
*M*	20.06	0.72	0.19	0.29	0.35	0.17	3.25	2.79	2.77	3.08	3.01	3.22
*SD*	1.83	0.45	0.39	0.46	0.48	0.37	0.63	0.76	0.78	0.73	0.71	0.69

*p ≤ 0.05. **p ≤ 0.01. ***p ≤ 0.001

### Measurement Models

A measurement model was estimated for each outcome to examine the fit of the latent constructs. Each model included the three latent constructs (near-peer social capital, peer social capital, and self-initiated social capital) as well as a latent construct of the outcome variable. All measurement models also included the following covariates: age, gender, race/ethnicity, and program. The measurement models for all three models showed good fit and all items had significant factor loadings (see Table 3).

**Table 3:** Fit Indices of Measurement Models

Measurement model	*χ*2	df	*p*-value	CFI	SRMR	RMSEA	Factor loadings range
Progress towards education and/or career goals	147.84	81	*p* < 0.001	0.985	0.021	0.031	0.65–0.94
Commitment to paying-it-forward	194.06	99	*p* < 0.001	0.981	0.023	0.034	0.63–0.95
Collective efficacy to change systems	126.48	81	*p* < 0.001	0.991	0.019	0.026	0.65–0.93

### Mediational Structural Equation Models

The structural equation models were specified to assess the association between emerging adults’ social capital with peers and near-peers and outcomes, including self-initiated social capital as a mediator. All three models controlled for age, gender, race/ethnicity, and program. The model for progress towards education and/or career goals, CFI = 0.980, SRMR = 0.021, and RMSEA = 0.031, commitment to paying-it-forward, CFI = 0.976, SRMR = 0.023, and RMSEA = 0.034, and collective efficacy to change systems, CFI = 0.989, SRMR = 0.019, and RMSEA = 0.026, showed good fit indices.

**Fig. 1:**
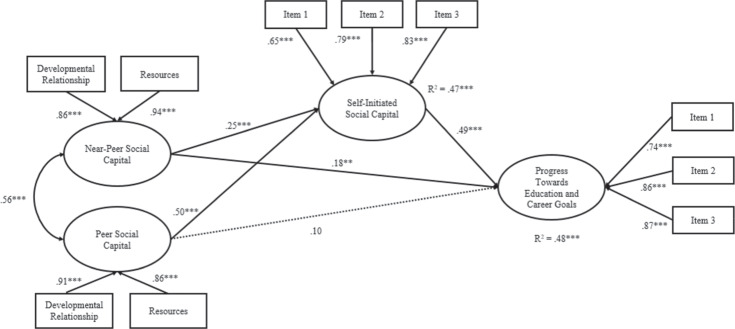
Mediation Model Depicting Self-Initiated Social Capital as a Mediator of the Relationship of Peer and Near-Peer Social Capital on Progress Towards Education and Career Goals. Standardized coefficients are presented. *p ≤ 0.05. **p ≤ 0.01. ***p ≤ 0.001

In the first model (see Fig. 1), near-peer social capital was significantly and directly associated with greater progress towards education and/or career goals (β = 0.18, *SE* = 0.07, *p* < 0.01). Near-peer social capital was also significantly associated with self-initiated social capital (β = 0.25, *SE* = 0.06, *p* < 0.001), which in turn, was significantly associated with greater progress towards education and/or career goals (β = 0.50, *SE* = 0.06, *p* < 0.001). The mediation utilizing bootstrapping supported the presence of a significant indirect effect from near-peer social capital (β = 0.122, *SE* = 0.036, *p* < 0.01; 95% bias corrected bootstrapped CI [0.07; 0.19]) to progress towards education and/or career goals through self-initiated social capital, providing support for partial mediation. Peer social capital was unrelated to progress towards education and/or career goals but was significantly associated with self-initiated social capital (β = 0.49, *SE* = 0.07, *p* < 0.001). The mediation analysis supported the presence of a significant indirect effect from peer social capital (β = 0.245, *SE* = 0.047, *p* < 0.001; 95% bias corrected bootstrapped CI [0.18; 0.34]) to progress towards education and/or career goals through self-initiated social capital, providing support for full mediation. The model explained 48% of the variance of progress towards education and/or career goals.

**Fig. 2:**
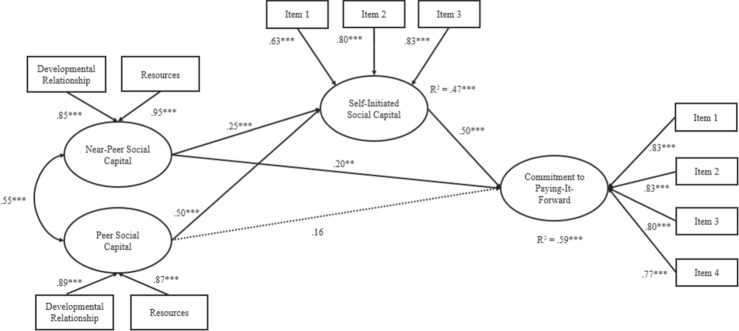
Mediation Model Depicting Self-Initiated Social Capital as a Mediator of the Relationship of Peer and Near-Peer Social Capital on Commitment to Paying-it-Forward. Standardized coefficients are presented. *p ≤ 0.05. **p ≤ 0.01. ***p ≤ 0.001

In the second model (see Fig. 2), a similar pattern emerged: near-peer social capital was significantly and directly associated with commitment to paying-it-forward (β = 0.20, *SE* = 0.06, *p* < 0.01). Near-peer social capital was also significantly associated with self-initiated social capital (β = 0.25, *SE* = 0.06, *p* < 0.001), which in turn, was significantly associated with greater commitment to paying-it-forward (β = 0.50, *SE* = 0.08, *p* < 0.001). There was a significant indirect effect from near-peer social capital (β = 0.123, *SE* = 0.032, *p* < 0.001; 95% bias corrected bootstrapped CI [0.07; 0.18]) to commitment to paying-it-forward through self-initiated social capital. Peer social capital was unrelated to commitment to paying-it-forward but was significantly associated with self-initiated social capital (β = 0.51, *SE* = 0.06, *p* < 0.001). There was a significant indirect effect from peer social capital (β = 0.255, *SE* = 0.053, *p* < 0.001; 95% bias corrected bootstrapped CI [0.18; 0.35]) to commitment to paying-it-forward through self-initiated social capital. The model explained 59% of the variance of commitment to paying-it-forward.

**Fig. 3:**
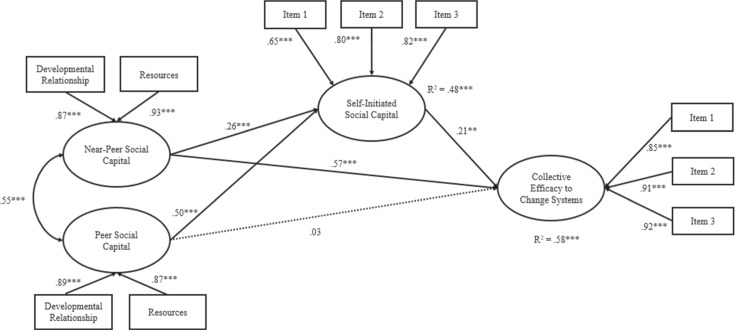
Mediation Model Depicting Self-Initiated Social Capital as a Mediator of the Relationship of Peer and Near-Peer Social Capital on Collective Efficacy to Change Systems. Standardized coefficients are presented. *p ≤ 0.05. **p ≤ 0.01. ***p ≤ 0.001

In the last model (see Fig. 3), the same pattern emerges again: near-peer social capital was significantly and directly associated with collective efficacy to change systems (β = 0.57, *SE* = 0.05, *p* < 0.001). Near-peer social capital was also significantly associated with self-initiated social capital (β = 0.26, *SE* = 0.06, *p* < 0.001), which in turn, was significantly associated with greater collective efficacy to change systems (β = 0.21, *SE* = 0.06, *p* < 0.01). There is a significant indirect effect from near-peer social capital (β = 0.05, *SE* = 0.02, *p* < 0.01; 95% bias corrected bootstrapped CI [0.03; 0.09]) to collective efficacy to change systems through self-initiated social capital. Peer social capital was unrelated to collective efficacy to change systems but was significantly associated with self-initiated social capital (β = 0.50, *SE* = 0.06, *p* < 0.001). There is a significant indirect effect from peer social capital (β = 0.107, *SE* = 0.036, *p* < 0.01; 95% bias corrected bootstrapped CI [0.05; 0.17]) to collective efficacy to change systems through self-initiated social capital. The model explained 58% of the variance of collective efficacy to change systems.

### Exploratory Multigroup Analysis by Race/Ethnicity

Multigroup analyses were performed to determine whether the proposed mediation models differed by emerging adults’ racial/ethnic identity. Unconstrained models demonstrated good fit to the data for all three outcomes (see Table 4). The unconstrained models were then compared to fully constrained models, where paths were constrained across racial/ethnic groups for each of the three outcomes. For both the progress towards education and/or career goals and the collective efficacy to change systems models, the χ2 difference test was not significant and revealed that the unconstrained model did not have a better fit to the data when compared to the constrained (i.e., more parsimonious) model. This suggests that the associations tested in the full model did not differ by emerging adults’ racial/ethnic identity. For the commitment to paying-it-forward outcome, the χ2 difference test between the constrained model and the unconstrained model was significant (see Table 4). A subsequent partially constrained model was then specified by constraining the mediated parameters and then comparing them to the fully unconstrained model. The χ2 difference test was also significant and revealed that the unconstrained model had a better fit to the data, suggesting that there are significant differences in the mediated parameters across different racial/ethnic groups. It was found that for emerging adults who identify as Asian or were in the Other group, near-peer social capital was not directly or indirectly associated with commitment to paying-it-forward. In contrast, near-peer social capital was directly associated with greater commitment to paying-it-forward for emerging adults who identify as Black (β = 0.530, *SE* = 0.141, *p* < 0.001) or Latinx (β = 0.270, *SE* = 0.110, *p* < 0.05). Peer social capital was unrelated to commitment to paying-it-forward for emerging adults who identify as Latinx. In contrast, peer social capital remained indirectly associated with greater commitment to paying-it-forward via self-initiated social capital among emerging adults who identified as Asian (β = 0.391, *SE* = 0.138, *p* < 0.001; 95% bias corrected bootstrapped CI [0.24; 0.59]), Black (β = 0.162, *SE* = 0.09, *p* < 0.05; 95% bias corrected bootstrapped CI [0.04; 0.39]), or were in the Other group (β = 0.323, *SE* = 0.15, *p* < 0.05; 95% bias corrected bootstrapped CI [0.11; 0.61]).

**Table 4:** Model Fit Indices for Race/Ethnicity Multigroup Structural Models

Model	*χ*2 (df)	*CFI*	*RMSEA*	*SRMR*	*χ*2 (df) diff
Progress towards education and/or career goals					
Unconstrained	395.92 (288)***	0.969	0.043	0.047	
Constrained	449.70 (339)***	0.968	0.040	0.073	53.8 (51)
Collective efficacy to change systems					
Unconstrained	358.64 (288)***	0.983	0.034	0.042	
Constrained	414.78 (339)***	0.981	0.033	0.073	56.14 (51)
Commitment to paying-it-forward					
Unconstrained	479.34 (354)***	0.969	0.041	0.051	
Constrained	557.92 (405)***	0.962	0.043	0.076	78.58 (51)**
Partially constrained	520.62 (369)***	0.962	0.045	0.071	41.28 (15)***

Mediation paths were constrained across racial/ethnic groups in the partially constrained models

*p ≤ 0.05. **p ≤ 0.01. ***p ≤ 0.001

### Exploratory Multigroup Analysis by Program Type

Multigroup analyses were also performed to determine whether the proposed mediation models differed by program type (i.e., workforce or education support program). Fully unconstrained models, in which all paths were allowed to vary across groups, were specified for each of the three outcomes and showed good fit to the data (see Table 5). The unconstrained models were then compared to fully constrained models, where paths were constrained across the two program types for each of the three outcomes. For all three outcomes, the χ2 difference test was significant and revealed that the unconstrained model had a better fit to the data when compared to the constrained model. Partially constrained models were then specified by constraining the mediated parameters and comparing them to the fully unconstrained model for each of the three outcomes. The χ2 difference test was significant and revealed that the unconstrained model had a better fit to the data when compared to the partially constrained models, suggesting that there are significant differences in the mediated parameters across the two program types.

**Table 5:** Model Fit Indices for Program Type Multigroup Structural Models

Model	*χ*2 (df)	*CFI*	*RMSEA*	*SRMR*	*χ*2 (df) diff
Progress towards education and/or career goals					
Unconstrained	229.89 (126)***	0.970	0.044	0.039	
Constrained	256.68 (141)***	0.966	0.044	0.068	26.79 (15)*
Partially constrained	246.43 (131)***	0.966	0.046	0.064	16.55 (5)**
Collective efficacy to change systems					
Unconstrained	194.80 (126)***	0.983	0.036	0.032	
Constrained	246.14 (141)***	0.974	0.042	0.121	51.34 (15)***
Partially constrained	229.55 (131)***	0.975	0.042	0.115	34.75 (5)***
Commitment to paying-it-forward					
Unconstrained	299.70 (156)***	0.964	0.047	0.037	
Constrained	336.11 (171)***	0.958	0.048	0.076	36.41 (15)**
Partially constrained	323.08 (161)***	0.959	0.049	0.070	23.38 (5)***

Mediation paths were constrained across program type (i.e., workforce or education support program) in the partially constrained models

*p ≤ 0.05. **p ≤ 0.01. ***p ≤ 0.001

Near-peer social capital had a significant direct (but not indirect) effect on progress towards education and/or career goals for participants in workforce support programs (β = 0.280, *SE* = 0.091, *p* < 0.01), whereas near-peer social capital had both a significant direct (β = 0.184, *SE* = 0.078, *p* < 0.05) and indirect effect (β = 0.159, *SE* = 0.047, *p* < 0.01; 95% bias corrected bootstrapped CI [0.09; 0.25]) among participants in the education support programs. Peer social capital had a significant indirect effect on progress towards education and/or career goals via self-initiated social capital for participants in both the workforce (β = 0.234, *SE* = 0.082, *p* < 0.01; 95% bias corrected bootstrapped CI [0.13; 0.40]) and education support programs (β = 0.258, *SE* = 0.058, *p* < 0.001; 95% bias corrected bootstrapped CI [0.17; 0.36]).

Near-peer social capital was unrelated to commitment to paying-it-forward among participants in workforce support programs, but was both directly (β = 0.259, *SE* = 0.066, *p* < 0.001) and (β = 0.134, *SE* = 0.039, *p* < 0.01; 95% bias corrected bootstrapped CI [0.08; 0.22]) associated with greater commitment to paying-it-forward among participants in education support programs. Peer social capital had a significant indirect effect on commitment to paying-it-forward via self-initiated social capital among participants in both workforce (β = 0.321, *SE* = 0.105, *p* < 0.01; 95% bias corrected bootstrapped CI [0.18; 0.53]) and education support programs (β = 0.217, *SE* = 0.066, *p* < 0.001; 95% bias corrected bootstrapped CI [0.12; 0.34]).

Near-peer social capital was directly associated with greater collective efficacy to change systems among participants in workforce support programs (β = 0.568, *SE* = 0.108, *p* < 0.001), but was both directly (β = 0.605, *SE* = 0.064, *p* < 0.001) and indirectly (β = 0.061; *SE* = 0.030, *p* < 0.05; 95% bias corrected bootstrapped CI [0.03; 0.12]) associated with greater collective efficacy to change systems among participants in education support programs. Peer social capital was unrelated to greater collective efficacy to change systems among participants in workforce support programs, but was indirectly so via self-initiated social capital among participants in education support programs (β = 0.097, *SE* = 0.049, *p* < 0.05; 95% bias corrected bootstrapped CI [0.03; 0.19]).

## Discussion

Workforce and education support programs are often designed to provide greater access to high-quality relationships and resources (i.e. social capital) that are believed to support emerging adults navigate education and career opportunities. Yet, little is understood about the mechanism through which social capital strengthens education and/or career outcomes and how different types of peer relationships contribute to these outcomes. The current study examined the pathway through which peer and near-peer social capital are associated with emerging adults’ progress towards education and/or career goals, commitment to paying-it-forward, and collective efficacy to change systems. The results showed support for a full mediational model, where peer social capital was associated with greater self-initiated social capital, and in turn, was positively associated with outcomes. A partial mediational model was found for near-peer social capital, such that near-peer social capital had a direct and indirect effect on all three outcomes. This suggests that both peer and near-peer social capital support young adults as they work towards their goals and may do so through different pathways. While the pattern of findings was fairly consistent, exploratory analyses suggest that some mediation effects may vary among emerging adults from different racial/ethnic backgrounds and across education and workforce support programs.

While a strong body of evidence shows that positive peer relationships and strong peer networks are associated with academic achievement (Berthelon et al., 2019) and a successful school-to-career transition (Ruschoff et al., 2018), the current study provides empirical insights on the mechanism through which peer social capital may strengthen these outcomes. Peer social capital did not have a direct effect on education and career outcomes, but did so indirectly through self-initiated social capital. Past research establishes the important role peers play in the provision of emotional and social support (Wang & Eccles, 2012), which is foundational for increasing young people’s agency and confidence both in the global sense and in utilizing their relationships and resources in pursuit of their goals (i.e., self-initiated social capital). For example, past research shows that strong peer networks are associated with: young people’s willingness to seek help (Menna & Ruck, 2004); feelings of collective efficacy, social responsibility, and civic engagement (Flanagan, 2013; Wray-Lake & Abrams, 2020); and, that greater peer social support is associated with greater social engagement (i.e., motivation to interact with others; Scanlon et al., 2020). Similarly, another study showed that peers’ efficacy beliefs were positively associated with young people’s engagement in job search activities (i.e., a greater number of applications completed) and indirectly associated with their job search outcomes (Ruschoff et al., 2018). Thus, peers may be uniquely situated to increase emerging adults’ self-initiated social capital, which is in turn strongly associated with education and career outcomes.

Unlike peer social capital, near-peer social capital was also directly associated with all three education and career outcomes. Because near-peers are slightly older and/or more experienced than program participants, they may serve in a more traditional, “institutional agent” role by providing greater access to valuable connections and resources than program peers (Stanton-Salazar, 2011). For example, near-peers may be able to provide more forms of instrumental support (e.g., skill-building, resume support, college navigation support) that directly impact emerging adults’ education and career outcomes. This is consistent with past research that shows a direct link between programs that provide cross-age peer mentoring relationships and positive social-emotional wellbeing and academic achievement (Karcher & Berger, 2017). In the day-to-day work and interactions in these programs, near-peers also serve as proximal role models for paying-it-forward by giving back the knowledge and opportunities that were created for many of them, and for engaging in collective efforts to ensure the systems that pave the way to education and career opportunities are more just and equitable.

It is also important to note that peer and near-peer social capital was not only associated with emerging adults’ progress towards education and/or career goals, but also their commitment to paying-it-forward and collective efficacy to change systems. This finding suggests that an added benefit of peer and near-peer social capital is that emerging adults with greater social capital are likely to go on and contribute to the wellbeing and social capital of others, including peers that are also working towards their education and career goals. This finding is consistent with the social capital concept of reciprocity, in which resources flow between actors (i.e., peers) in both directions (Torche & Valenzuela, 2011). It is likely that as young adults experienced strong developmental relationships with peers and near-peers and increased access to resources, they were also motivated to share these newfound resources with other peers; generating a sense that by joining forces they had the potential to help change education and employment systems. The ripple effects of peer and near-peer social capital across social networks and institutional justice is ripe for further study.

The current study examined *developmental* peer and near-peer relationships as a component of social capital. It was theorized that developmental relationships may provide emerging adults with more relational resources than other high-quality, caring relationships (Scales et al., 2020). Developmental relationships include elements that go beyond caring, such as high expectations, providing instrumental forms of support (e.g., information sharing, skill development), sharing power (e.g., involvement in decision-making, opportunities to lead), and expanding possibilities (e.g., introductions to key contacts and access to the college and workforce norms; Pekel et al., 2018). These types of elements, especially provide support, share power, and expand possibilities, may help create not just “bonding” social capital, but also “linking” social capital that helps emerging adults navigate postsecondary institutions and pathways that they may not otherwise have access to. This is consistent with findings from recent research that shows developmental relationships with adults (i.e., educators, mentors) is an important component of social capital that is strongly linked with positive academic outcomes (Scales et al., 2021) and work readiness (Boat et al., 2021) among adolescents and emerging adults. The current study extends this research by showing how social capital resulting from peer and near-peer developmental relationships contributes to positive education and career outcomes. Future research should continue to examine which elements of a developmental relationship are most important for strengthening social capital and postsecondary outcomes.

The value of peer and near-peer social capital may be especially underestimated among young adults of color. Studies have found that socializing with peers is associated with higher levels of academic persistence among Latinx students (Otero et al., 2007) and college students tend to rely on peer networks of the same ethnicity when seeking assistance in adapting to the college environment (Eunyoung, 2009). Furthermore, students of color often provide important resources for each other such as modeling pro-academic behaviors and norms, and supporting ethnic identity development (Stanton-Salazar, 2004). The current sample was predominantly made up of young adults of color who may have especially benefited from developing strong relationships with peers and near-peers in their program; all of whom were working towards education and career achievements and many of whom identified as people of color and/or as coming from backgrounds similar to those of program participants. More encouraging still, the current study illustrates how peer and near-peer social capital are positively associated with young adults’ commitment to paying-it-forward and collective efficacy to change education and employment systems. This suggests that the social capital derived from these peer relationships may have positive effects on other young adults of color as well as education and employment systems.

Exploratory multigroup analyses did, however, suggest that the mediation paths between peer and near-peer social capital and outcomes may vary among different racial/ethnic groups. Near-peer social capital was unrelated to commitment to paying-it-forward for emerging adults who identified as Asian, but was directly related to greater commitment to paying-it-forward for emerging adults who identified as Black or Latinx. Unfortunately, the current study does not have information on the racial/ethnic identity of near-peers, themselves. However, many of the near-peers were past program participants and likely identify as a person of color. There is some research that suggests mentoring relationships where mentees are paired with mentors who identify as the same racial/ethnic background may forge stronger, longer lasting relationships (Raposa et al., 2019). Perhaps, more emerging adults who identified as Black or Latinx were engaged with near-peers who identified as the same racial/ethnic background, and thus had a stronger effect on one’s commitment to paying-it-forward. Additionally, peer social capital was unrelated to commitment to paying-it-forward among emerging adults who identified as Latinx, but was indirectly so for emerging adults who identified as Asian or Black. It is unclear why this finding emerged; it is possible that other factors are important for contributing to Latinx emerging adults’ commitment to paying-it-forward such as family social capital. One such study, for example, documents a strong reciprocal relationship between Latino male college students and their extended families (Patrón, 2020). Future research should continue to examine multiple sources of social capital (e.g., family members, natural mentors, community members) and how these relationships contribute to one’s motivation to reciprocate social capital with others.

### Practice Implications

This study has important implications for young adult-serving organizations that support emerging adults through education and career pathways. The study demonstrates the importance of understanding the web of relationships in a young person’s life, as different relationships may provide different forms of support and/or be related to outcomes through different pathways. This study, in particular, emphasizes the role of peers and near-peers in promoting emerging adults’ education and career outcomes. Given this finding, organizations that serve emerging adults, especially young people of color, may incorporate practices and programs that strengthen peer networks and developmental relationships as a way to help support young people’s life success. A recent report, for example, highlights a number of youth- and young adult-serving organizations and practical ways in which these programs support peer relationships, including utilizing virtual platforms for peers to connect, providing one-on-one coaching or mentoring from near-peers, developing shared rituals to foster a strong sense of community, providing peer tutoring opportunities, convening peers to talk about their sense of purpose, identity, and future goals, among many others (Waite, 2021). Future research should examine which of these practices and tools may be most impactful for fostering social capital among peers and near-peers.

Consistent with youth development theories, findings in the current study show that emerging adults are active agents in their own development and actively use their social capital to reach goals (Schwartz & Rhodes, 2016; Lerner et al., 2015). Thus, in addition to finding practical ways to nurture strong and resource-rich peer-to-peer and near-peer developmental relationships, organizations can further maximize their impact by strengthening young people’s agency and ability to utilize their peer networks as they work towards their life goals. This can be done with intention in several different ways; however, at the heart of each of them is a need to empower emerging adults with the confidence and skills needed to agentically capitalize on the resources available to them. This work might take the shape of adding content to program activities that allows young adults to practice, for example, making a “cold call” to establish a new connection, or asking someone in their network for assistance or to forge a new connection on their behalf. It might also take the shape of a reflective assessment where program participants map their social networks, along with the gaps and opportunities within their network, then—with support for peers, near-peers, and/or adult staff—make a concrete action plan to activate their connections. Some interventions and programs have started to take this approach with success. Youth-initiated mentoring, for example, shows that youth are able to effectively select mentors who go on to serve as valuable sources of support (Schwartz et al., 2016). Other studies have found that young people who discussed intentional initiation behaviors often went on to develop greater connection in their relationships with supportive adults (Futch Ehrlich et al., 2016).

Moreover, exploratory multigroup models suggest that the mechanism through which peer and near-peer social capital are associated with positive education and career outcomes may vary by program type. While all five programs in the current study supported emerging adults with both education and career goals to some extent, two of the programs primarily served current college students and three of the programs primarily served recent college graduates. Given the different developmental stages of these two groups, two of the programs were more focused on current educational goals and the other programs were focused on providing more workforce skill development. Both peer and near-peer social capital tended to be positively associated with all three outcomes across both education and workforce support programs, yet the pathway through which peer and near-peer social capital was associated with these outcomes sometimes varied. The most consistent variation was the effect that near-peer social capital had on outcomes across the two program types. Near-peer social capital was indirectly associated with all three outcomes (via self-initiated social capital) among individuals in education support programs, whereas near-peer social capital was only directly associated with progress towards education and/or career goals and collective efficacy to change systems among program participants in workforce support programs. While these findings are exploratory and should be interpreted with caution, they do suggest that the mechanism through which near-peer social capital is associated with young adults’ education and career outcomes varies across program type. This variation may be due to differences in the goal young adults are working towards. Within workforce support programs, near-peer social capital may have a more direct impact, as near-peers make connections and share information that helps young adults secure an interview or land a job. Whereas near-peer social capital may at times play a more indirect role among young adults working towards an education goal. Near-peers in these types of programs may provide more forms of emotional and social support to help young adults build the confidence and agency needed to utilize their social capital in pursuit of educational goals.

### Limitations and Future Directions

The current study should be interpreted in light of its limitations. The survey was sent to a large number of program participants, of which only a small percentage (31%) chose to participate in and complete the study survey. As such, non-response bias may be present. The sample was also primarily drawn from one program. While the analyses controlled for programs within each of the models, it is possible that there are differences in the program models and/or samples that influenced the results. Exploratory multigroup analysis of program type provides some evidence for this, and suggest that there are at least differences in how mediation effects operated among education and workforce support programs. Several racial/ethnic identities needed to be collapsed in order to run multigroup models and not all racial/ethnic identities were represented. Given that the exploratory multigroup analyses showed that mediation effects may vary across both racial/ethnic groups and program type, it will be important for future studies to continue to examine how these pathways may vary across more diverse samples of emerging adults including young adults from different socioeconomic backgrounds and in different types of programs. This study is cross-sectional and thus the temporal ordering of the associations between program peer and near-peer social capital, self-initiated social capital, and education and career outcomes cannot be confirmed. For example, it is possible that self-initiated social capital precedes peer and/or near-peer social capital, which are in turn the more proximal indicators of education and career outcomes. Future research should build on this work testing the mediational model longitudinally to confirm the pathway through which peer and near-peer social capital are associated with education and career outcomes. Progress towards education and career goals as well as the other two outcomes, were assessed using self-reported measures. Future research should assess whether similar results emerge when using more concrete assessments of young adults’ progress towards their education and/or career goals such as education and career attainment (e.g., college graduation rates, successfully securing first job post-graduation).

## Conclusion

Social capital and youth development theorists have consistently argued that strong relationships are critical for emerging adults’ postsecondary success, and that strong workforce and education support programs that provide access to this type of social capital is one avenue for facilitating success. The current study shows how peer and near-peer social capital, in particular, can help young adults of color make progress towards their education and/or career goals, while also increasing their commitment to paying-it-forward and working together to improve systems that, too often, exclude people of color. Moreover, this study demonstrates one mechanism through which peers and near-peers do this: by building emerging adults’ skills and agency to mobilize and expand their social capital. Specifically, a full mediational model was supported in which peer social capital was positively associated with greater self-initiated social capital, and in turn emerging adults’ progress towards education and career goals, commitment to paying-it-forward, and collective efficacy to change systems. A partial mediational model showed that near-peer social capital had a direct and indirect effect on all three outcomes. The actionable knowledge generated from this study points to clear and malleable features of young adult-serving organizations that can be leveraged to positively shape the postsecondary trajectories of participants, *and* feed a reciprocal cycle of social capital generation that has the potential to redress unjust systems by creating viable pathways for all young adults to pursue their education and career goals.
